# I want it all, and I want it now

**DOI:** 10.1007/s12471-020-01368-3

**Published:** 2020-01-21

**Authors:** R. J. de Winter

**Affiliations:** Department of Cardiology, Amsterdam UMC, Amsterdam, The Netherlands

Primary percutaneous coronary intervention (PCI) in patients with ST-elevation myocardial infarction (STEMI), i.e. mechanical reperfusion therapy as soon as possible by opening the infarct-related occluded epicardial coronary vessel, has been shown to reduce mortality. Approximately 50% of STEMI patients present with multivessel disease, which is associated with worse prognosis. Guidelines recommend complete revascularisation, but earlier studies have shown mixed results. In recent years, several randomised controlled studies and meta-analyses have demonstrated that multivessel PCI in STEMI patients was associated with a significant reduction in the composite of death, non-fatal myocardial infarction, repeat revascularisation or stroke [1, 2]. Importantly, when non-culprit lesions are selected based on fractional flow reserve (FFR) measurements, around 30% of angiographically significant lesions qualify as haemodynamically non-significant. In addition, the difference in outcome in the larger contemporary trials was mainly driven by a difference in repeat revascularisation [3]. In the COMPLETE trial, complete revascularisation was superior to culprit-lesion-only PCI in reducing the risk of cardiovascular death or myocardial infarction, as well as the risk of cardiovascular death, myocardial infarction, or ischaemia-driven revascularisation [4]. In the Netherlands, the ongoing iMODERN study (NCT03298659) will answer the question whether instant wave-free ratio (iFR) guided complete revascularisation during the index hospitalisation is superior to deferred cardiac magnetic resonance guided additional revascularisation.

In patients with non-ST-elevation acute coronary syndromes, early revascularisation is recommended for “high-risk” patients with a GRACE risk score >140 or a positive cardiac troponin. The question whether complete revascularisation in non-STEMI patients is associated with clinical benefit has not been studied extensively, although a large registry showed a benefit associated with single-stage complete coronary revascularisation [5]. In this issue of the Netherlands Heart Journal, Pustjens and colleagues report the rationale and design of the South Limburg Myocardial Infarction (SLIM) trial [6]. The SLIM trial is an investigator-initiated, prospective, multicentre, randomised controlled trial that compares FFR-guided complete revascularisation during the index procedure with usual care in non-STEMI patients with multivessel disease. A total of 414 patients will be randomised in a 1:1 fashion and the trial is scheduled to enrol patients over a period of 3 years. The primary endpoint is the composite of all-cause mortality, non-fatal myocardial infarction, and any revascularisation and stroke (MACE) at 12 months. Secondary endpoints include follow-up at 24 and 36 months.

Several design features stand out:

- Similar to STEMI trials, a verbal informed consent is obtained during the index procedure after successful PCI of the culprit lesion, followed by a written informed consent prior to leaving the catheterisation laboratory.
- It will be of utmost importance to correctly identify the culprit lesion. Thus in the trial, the use of anatomic (intravascular ultrasound or optical coherence tomography) or functional (FFR) imaging modalities are used to assess the culprit lesion.
- Stenosis in non-infarct-related artery (non-IRA) amenable for PCI treatment (operator’s decision): In the ischaemia-driven complete revascularisation group, all flow-limiting lesions (FFR <0.80) will be treated with PCI and stenting.
- In the usual care group, the procedure will stop after PCI of the culprit artery and the patient will be referred to his/her treating cardiologist and/or heart team. They will decide whether or not (ischaemia-driven) staged PCI of the non-IRA should take place. The following treatment options are possible: (1) FFR- or iFR-guided PCI of the non-IRA, (2) additional non-invasive tests, (3) symptom-driven PCI of the non-IRA, or (4) optimal medical therapy. If the treating cardiologist (after consulting the heart team) decides to perform the non-IRA PCI revascularisation, then such treatment should take place within 6 weeks of the primary PCI in order to count as a scheduled staged PCI procedure.
- An interim analysis is scheduled at six months after 150 patients have been included.

The trial was scheduled to start in June 2018, therefore perhaps we can expect the first interim analysis with the first 150 patients in due course. The sample size of 414 patients has been chosen based on the SMILE trial, expecting the incidence of the primary endpoint in the culprit-only treated patients at 12 months to occur in 20% of patients compared with 10% in the multivessel PCI group [5]. This absolute 10% difference would demonstrate superiority of the multivessel PCI strategy. However, this may be optimistic, given the fact that the approximately 20% major adverse cardiac events (MACE) in the SMILE trial was mainly driven by an unexpected incidence of target vessel revascularisation (8.3% vs 15.2%), which predominantly occurred between 6 and 12 months. Perhaps the investigators can use the interim analysis to look at the effect estimate and the actual incidence of MACE. When the difference in MACE between the two randomised treatment strategies is less than the anticipated 10%, an increase of the sample size may be anticipated.

In summary, this will be a very interesting, investigator-initiated study and the results will be eagerly awaited. Worst case scenario (in my mind) would be that complete revascularisation during the index procedure in non-STEMI patients prevents PCI in non-culprit lesions at a later timepoint without a difference in other hard endpoints such as death, myocardial infarction or stroke. Best case scenario would be that FFR-guided complete revascularisation is superior in reducing MACE, and at the same time shows a reduction in recurrent hospital admission, the need for additional ischaemia testing and overall cost.
